# Provider Education about Glaucoma and Glaucoma Medications during Videotaped Medical Visits

**DOI:** 10.1155/2014/238939

**Published:** 2014-04-24

**Authors:** Betsy Sleath, Susan J. Blalock, Delesha M. Carpenter, Kelly W. Muir, Robyn Sayner, Scott Lawrence, Annette L. Giangiacomo, Mary Elizabeth Hartnett, Gail Tudor, Jason Goldsmith, Alan L. Robin

**Affiliations:** ^1^UNC Eshelman School of Pharmacy and Cecil G. Sheps Center for Health Services Research, University of North Carolina at Chapel Hill, CB No. 7590, Chapel Hill, NC 27599-7590, USA; ^2^Division of Pharmaceutical Outcomes and Policy, UNC Eshelman School of Pharmacy, University of North Carolina at Chapel Hill, Chapel Hill, NC 27599, USA; ^3^Department of Ophthalmology, School of Medicine, Duke University, Durham, NC 27710, USA; ^4^Health Services Research and Development, Durham VA Medical Center, Durham, NC 27710, USA; ^5^Glaucoma Service and Research Center, UNC Kittner Eye Center, University of North Carolina at Chapel Hill, Chapel Hill, NC 27599, USA; ^6^Department of Ophthalmology, Emory University School of Medicine, Atlanta, GA 30322, USA; ^7^Department of Ophthalmology and Visual Sciences, John A. Moran Eye Center, University of Utah, Salt Lake City, UT 84132, USA; ^8^Department of Science and Mathematics, Institutional Research, Husson University, Bangor, ME 04401, USA; ^9^Department of International Health, Bloomberg School of Public Health, Johns Hopkins University, Baltimore, MD 21209, USA; ^10^Department of Ophthalmology, School of Medicine, Johns Hopkins University, Baltimore, MD 21209, USA

## Abstract

*Objective*. The purpose of this study was to examine how patient, physician, and situational factors are associated with the extent to which providers educate patients about glaucoma and glaucoma medications, and which patient and provider characteristics are associated with whether providers educate patients about glaucoma and glaucoma medications. *Methods*. Patients with glaucoma who were newly prescribed or on glaucoma medications were recruited and a cross-sectional study was conducted at six ophthalmology clinics. Patients' visits were videotape recorded and patients were interviewed after visits. Generalized estimating equations were used to analyze the data. *Results*. Two hundred and seventy-nine patients participated. Providers were significantly more likely to educate patients about glaucoma and glaucoma medications if they were newly prescribed glaucoma medications. Providers were significantly less likely to educate African American patients about glaucoma. Providers were significantly less likely to educate patients of lower health literacy about glaucoma medications. *Conclusion*. Eye care providers did not always educate patients about glaucoma or glaucoma medications. *Practice Implications*. Providers should consider educating more patients about what glaucoma is and how it is treated so that glaucoma patients can better understand their disease. Even if a patient has already been educated once, it is important to reinforce what has been taught before.

## 1. Introduction 

Glaucoma is one of the leading causes of blindness and visual disability. An estimated 1.5 million Americans suffer from glaucoma while approximately 120,000 of them have been blinded by the disease. Between 9 and 12% of all blindness in the United States is attributed to glaucoma [1]. The primary goal of glaucoma treatment is to reduce intraocular pressure [2, 3]. Consistently taking intraocular pressure-lowering glaucoma medications can significantly reduce the progression of glaucoma [4, 5].

Little is known about ophthalmologist-patient communication during glaucoma visits [6]. Prior work has linked inconsistent glaucoma follow-up with unfamiliarity with the duration of glaucoma treatment and lack of knowledge of the permanency of glaucoma-induced vision loss [7]. In a prior study, researchers found that, even though most patients reported their ophthalmologist was their primary source of information about glaucoma, 15% reported that their ophthalmologist told them either “not much” or “nothing” about glaucoma [8].

Similarly, little is known about what ophthalmologists actually tell patients about glaucoma during visits. One prior study interviewed patients about what their ophthalmologists discussed during visits [6], but they did not videotape the patients' visits to examine actual communication. The researchers found that when patients were asked “how much of what you know about glaucoma did you hear first from your doctor?” 32% responded “all that they know,” 30% said “most of what they know,” 32% said “some but not much,” and 5.3% said “nothing.” We need to have a better understanding of what actually transpires between an ophthalmologist and a patient during an encounter. Videotapes offer an impartial method of assessing what actually transpires.

To our knowledge, no prior study has used videotape recordings to examine the doctor-patient communication to assess the extent to which ophthalmologists provide education about glaucoma medications during visits and how this is associated with various patient characteristics. When evaluating antidepressant therapy, two prior studies found that providers were more likely to give patients information if they were newly prescribed an antidepressant for the first time versus already being on one [9, 10]. In one study, physicians were most likely to give the following type of information about antidepressants: purpose (27.5%), dose (22.5%), supply (15%), which antidepressant to take (15%), and timing (12.5%) [9].

Prior audiotaped examinations of the doctor interactions with patients with asthma have found that providers educated families about medications during 61% of visits [11]. Providers educated their patients about medications most often in the following areas: (a) frequency/timing of use (37%), (b) strength/dose (32%), and (c) purpose (30%). They provided education regarding side effects during only 7% of encounters [11]. Research is needed to better understand in what areas ophthalmologists provide education for patients about glaucoma and glaucoma medications.

The theoretical rationale for this study is the ecologic model of communication in medical consultations [12, 13]. This model hypothesizes that the way patients communicate with physicians is influenced by personal, physician, and contextual factors [12, 13].* Personal factors* could be the patient's gender, race, age, and health literacy; [13]* provider factors* could be age, gender, and race; and* contextual or situational factors* could be whether the visit is a follow-up or an initial visit [12, 13].

Therefore, the purpose of this study was to apply the ecologic model of communication in medical consultations [12, 13] to examine how patient (age, gender, race, literacy, and years of education), physician (age, gender, and race), and situational (whether glaucoma medications are prescribed for the first time) factors are associated with (a) the extent to which providers educate patients about glaucoma and glaucoma medications and (b) which patient and provider characteristics are associated with whether providers educate patients about glaucoma and glaucoma medications.

## 2. Patients and Methods

### 2.1. Procedure

Cross-sectional study took place at six geographically distinct ophthalmology clinics located in the United States. Two sites were private offices and four were affiliated with academic ophthalmology departments. Patients were enrolled between 2009 and 2012. Eligibility criteria included having the ability to speak and read English, having a diagnosis of glaucoma, and being at least 18 years of age. At each site, clinic staff referred eligible patients to research assistants who were based at the clinics. Written patient and provider consent was obtained. Providers completed a short demographic questionnaire after providing consent. The patient's medical visit was videotape recorded. Patients were interviewed immediately after their medical visits. The study was approved by the University of North Carolina Institutional Review Board, was performed in accordance with the tenants of the Treaty of Helsinki, and was HIPAA compliant.

### 2.2. Measurement

#### 2.2.1. Patient, Provider, and Situational/Contextual Measures

Patient age was measured as a continuous variable. Self-reported patient race was measured as a categorical variable (White, African American, Asian, Native American, and Hispanic) and then recoded into African American and non-African American. The majority of the non-African American patient sample was White (91%). Gender was measured as a dichotomous variable. The number of glaucoma medications a subject was taking was recorded.

Each subject received the rapid estimate of adult literacy in medicine (REALM). This is a validated, rapid screening instrument designed to identify patients who have difficulty reading common medical and lay terms that are routinely used in patient education materials [14]. We chose the REALM because it has high face validity and high criterion validity, it has been well received by patients, and it only takes two to three minutes to administer and score [14]. Patient scores on the REALM correspond to reading levels (score of 0–60 = eighth grade and below and 61–66 = ninth grade and above).

Physician age was measured as a continuous variable and physician gender was measured as a dichotomous variable. Self-reported physician race was measured as a categorical variable (White, African American, Asian, Native American, and Hispanic). We also examined whether gender and racial concordance between the provider and the patient influenced provider education, but it was not significantly associated with provider education about glaucoma or glaucoma medications, so it was not included in our analyses. The situational factor we measured was whether the patient was prescribed glaucoma medication for the first time during the medical visit or was already on glaucoma medication prior to the medical visit.

#### 2.2.2. Communication Measures

All medical visit videotapes were transcribed into text verbatim with identifiers removed. A detailed coding tool to assess communication was developed over a one-year period. The areas for education about glaucoma and about glaucoma medications were developed using prior literature and input from the pharmacists and ophthalmologists on the study team [6–8]. The transcripts were reviewed by a research assistant who met twice a month with the investigators to develop and refine the coding rules.

Using the coding tool for transcribed medical visits, coders recorded whether the provider educated the patient in the following areas about glaucoma: (a) physical changes with glaucoma and/or how to manage these changes, (b) emotional changes with glaucoma and/or how to manage these changes, (c) diagnosis, (d) family history, (e) goals of treatment, (f) how to problem solve, (g) intraocular pressure, (h) likelihood of long-term therapy, (i) ways to manage glaucoma other than with medications, and (j) prognosis.

Coders also recorded whether the provider educated the patient in the following areas about glaucoma medications: (a) adherence and adherence strategies, (b) amount/dose, (c) cost/insurance, (d) eyelid closure and nasolacrimal occlusion when applying topical medications, (e) fear/concerns/barriers, (f) frequency of use, (g) generic/brand, (h) how well medication is working, (i) how to administer, (j) side effects, (k) importance of use, (l) last time drops were used, (m) length of use, (n) name of medication, (o) nonglaucoma medications, (p) purpose, (q) supply, and (r) which eye to instill the drops.

Two clinics had fellows examine some of the enrolled patients while two other clinics had ophthalmic technicians examine some of the enrolled patients. Informed consent was obtained from these providers as well. If any one of these healthcare providers, including the physician, educated the patient, it was counted as education in the categories discussed above.

Three research assistants coded 25 of the same transcripts throughout the study period to assess inter-coder reliability which was calculated using inter-rater correlations. Inter-rater reliability was 0.76 for whether the physician provided education about glaucoma and was 0.88 for whether the physician provided education about glaucoma medications to the patient.

#### 2.2.3. Analysis

We set the a priori level of statistical significance at *P* < 0.05. First, we ran descriptive statistics. Second, we examined the bivariate relationships between variables using Pearson correlation coefficients, chi-square statistics, and *t*-tests as appropriate. We then examined how whether the patient was newly prescribed glaucoma medication on the day of the visit was associated with the glaucoma and glaucoma medication education areas using Pearson chi-square.

We conducted generalized estimating equations (GEE) to examine how patient's age, gender, race, and health literacy, whether the patient was newly prescribed glaucoma medication on the day of the visit, physician age, and physician gender, were associated with (a) whether the physician provided any education about glaucoma and (b) whether the physician provided any education about glaucoma medications. Physician race could not be included in the multivariable analysis because we only had one non-White physician.

## 3. Results

Fifteen physicians who cared for glaucoma patients agreed to participate in the study; one physician refused to participate for a participation rate of 94%. Fourteen physicians were White and one was African American. Ten physicians were male (66.7%). Physician age ranged from 26 to 66 years (mean 40.8 years, standard deviation 11.7 years).

Eighty-six percent of eligible patients participated in the study.  Table 1 presents the patient demographics. Forty-one percent of the sample was male and 35.5% were African American. Eighteen percent of patients were prescribed glaucoma medications for the first time.

Providers educated patients about one or more glaucoma medication areas during 74% of visits.  Table 2 presents the extent to which the providers educated the patients about their medications in different areas. When patients were newly prescribed glaucoma medications, the areas that providers educated them about most often included (a) side effects (80%), (b) purpose (45%), (c) adherence and adherence strategies (39%), (d) frequency of use (37%), (e) which eye to use the medicine in (33%), and (f) how to administer the medicine (26%). Providers only educated 16% of patients newly prescribed glaucoma medications on the amount/dose to use, 16% about the importance of use, and approximately 10% on the name of the medication.

As shown in Table 2, providers were significantly more likely to educate patients who were newly prescribed glaucoma medications than patients already on glaucoma medications in the following areas: (a) adherence and adherence strategies, (b) amount/dose, (c) frequency of use, (d) how to administer, (e) side effects, (f) importance of use, and (g) purpose of the medications. For patients who continued on glaucoma medications, providers were most likely to provide education in the following areas: (a) frequency of use (15.8%), (b) side effects (15.4%), and (c) adherence and adherence strategies (13.6%).

Providers educated patients about one or more glaucoma areas during 63% of visits.  Table 3 presents the extent to which providers educated the patients about glaucoma in specific areas. When patients were started on glaucoma medications for the first time, providers were most likely to educate patients in the following areas: (a) diagnosis (60.8%), (b) goals of treatment (56.9%), (c) intraocular pressure (56.9%), and (d) physical changes that can occur with glaucoma and/or how to manage these changes (52.9%). Education about the emotional changes that can occur with glaucoma and how to manage these changes only occurred during one visit.

As shown in Table 3, providers were significantly more likely to educate patients who were newly prescribed glaucoma medications than patients already on glaucoma medications in the following areas: (a) physical changes that can occur with glaucoma, (b) diagnosis, (c) goals of treatment, (d) how to problem solve, and (e) likelihood of long-term therapy. For patients who continued on glaucoma medication, providers were most likely to provide education in the following areas: (a) intraocular pressure (46.5%), (b) physical changes that can occur with glaucoma (26.3%), and (c) prognosis (25.9%).

Table 4 presents the generalized estimating equation results predicting whether providers educated patients about glaucoma medications during visits. Older physicians were significantly more likely to provide education about glaucoma medications than younger physicians (odds ratio = 1.04, 95% confidence interval = 1.01, 1.07). Providers were significantly less likely to provide glaucoma medication education to patients with lower health literacy (odds ratio = 0.38, 95% confidence interval = 0.18, 0.79). Providers were significantly more likely to provide glaucoma medication education to patients who were prescribed glaucoma medications for the first time during the visit than to patients who were already on glaucoma medications (odds ratio = 7.26, 95% confidence interval = 3.6, 14.6).

Table 4 also presents the generalized estimating equation results predicting whether providers educated patients about glaucoma during visits. Providers were significantly less likely to educate African American patients about glaucoma than non-African American patients during visits (odds ratio = 0.47, 95% confidence interval = 0.34, 0.66). Providers were significantly more likely to provide glaucoma education to patients who were prescribed glaucoma medications for the first time during the visit than to patients who were already on glaucoma medications (odds ratio = 5.23, 95% confidence interval = 2.1, 13.4).

## 4. Discussion and Conclusion

### 4.1. Discussion

Ophthalmologists educated their patients about glaucoma during 74% of visits. Education about the relevance of intraocular pressure occurred during only 57% of visits where glaucoma medications were prescribed for the first time and 47% of visits where the patient had already been placed on a medication during a prior visit. Providers should consider educating patients about the importance of intraocular pressure reduction during every visit to assist in reinforcing the importance of continued therapy.

Providers educated about the physical changes that occur with glaucoma during 53% of visits, the likelihood of long-term therapy during 24% of visits, and the goals of treatment during 57% of the visits of the patients who were prescribed glaucoma medications for the first time. Providers educated continued users even less often in these areas. Providers should consider educating more patients about these important areas so that glaucoma patients better understand their disease. Even if a patient has already been educated once, it is important to reinforce what was taught before.

As predicted by the ecological model of communication [12, 13], we found that a* contextual factor* (a patient being prescribed a glaucoma medication for the first time versus being a continued user) was significantly associated with provider education about glaucoma. Specifically, patients who were newly prescribed glaucoma medications during the visit were significantly more likely to receive education about glaucoma. Additionally, patient race was significantly associated with provider education about glaucoma.

Providers were significantly less likely to educate African American patients than non-African American patients about glaucoma. This is an important finding because glaucoma is the leading cause of irreversible blindness among the African American population [15]. Also, prior work has found that African Americans are less adherent to their glaucoma medications than White patients. [16–19] Providers should make sure to educate patients equally from all racial backgrounds. Other medical subspecialties have also found differences in care given among races [20–25]. This is consistent with other studies looking at racial disparities both in causes of visual disabilities and testing [26].

Providers educated about the emotional changes that occur with glaucoma during only one visit. Having glaucoma and having to use eye drops can be potentially stressful to patients; thus, provider discussions of emotional changes may help patients feel more prepared to deal with this stress. Providers should consider educating about the emotional changes that occur with glaucoma and assessing whether patients might have depressive symptoms or might be anxious about having the disease or having to use eye drops.

Providers educated patients about their glaucoma medications during 63% of visits. As predicted by the ecological model of communication [12, 13], we found that a* contextual factor* (a patient being prescribed a glaucoma medication for the first time versus being a continued user) was significantly associated with provider education about glaucoma medications. Specifically, patients who were newly prescribed glaucoma medications during the visit were significantly more likely to be educated about glaucoma medications. Additionally, patient literacy was significantly associated with provider education about glaucoma medications. Providers were significantly less likely to educate patients who read at an eighth grade level or below about their glaucoma medications. This is an important finding because patients with low health literacy are the ones who especially need education about their glaucoma medications. Future research should examine whether this is because providers believed that patients with lower literacy would be less likely to understand the information.

Providers educated the patients about the purpose of the medication during 45% of visits, frequency of use during 37% of visits, and how to administer the drops during 26% of visits of patients who newly started on glaucoma medications during the visit. Providers educated even fewer continued users of glaucoma medications in these areas. These areas are important when it comes to patients learning how to properly use their glaucoma medications. Studies with asthma patients have shown that patient medication technique deteriorates with time. [27, 28] This could also be the case with instilling eye drops. Thus, providers may want to periodically assess patients' eye drop technique.

Providers educated about the importance of adherence to medications during only 39% of visits where medications were prescribed for the first time and 14% of visits where medications were continued. Providers should educate about the importance of adherence to glaucoma medications during all visits especially since nonadherence to glaucoma medications may lead to worse clinical outcomes for glaucoma patients.

This study has several limitations. Providers and patients both knew the visit was being recorded, but they did not know the study hypotheses. Selection bias could be another limitation since the ancillary staff did not track the characteristics of the few patients who declined to speak with the research assistant to learn more about the study. Additionally, our coders counted the patient being educated about glaucoma or glaucoma medications during visits regardless of whether a physician, technician, or fellow provided it. A limitation is that we coded the data this way so we cannot separate out physician, technician, and fellow provision of education. Also, since the examination rooms were periodically dimmed during the medical visit, it was difficult to consistently observe nonverbal communication between the healthcare provider and the patient. Thus, we did not include nonverbal communication in the analysis. Despite these limitations, the study presents new information on the extent to which providers educated patients about glaucoma and glaucoma medications and highlights several areas where education can be improved.

### 4.2. Conclusion

Eye care providers were less likely to educate African American patients about glaucoma and they were less likely to educate patients of lower health literacy about glaucoma medications. Providers were significantly more likely to educate patients about glaucoma and glaucoma medications if they were newly prescribed glaucoma medications.

### 4.3. Practice Implications

Providers should consider educating more patients about what glaucoma is and how it is treated so that glaucoma patients can better understand their disease. Providers educated about the purpose of the medication, frequency of use, and how to administer the drops during less than 50% of visits of patients who newly started on glaucoma medications during the visit. Providers educated even fewer continued users of glaucoma medications in these areas. These areas are important when it comes to patients learning how to properly use their glaucoma medications. Even if a patient has already been educated once, it is important to reinforce what has been taught before.

## Figures and Tables

**Table 1:** Subject characteristics (*N* = 279).

	Number (%)
Gender	
Male	144 (40.9)
Female	165 (59.1)
Race	
African American	99 (35.5)
Non-African American	179 (64.2)
Newly prescribed glaucoma medications at visit or was on glaucoma medication before visit	
Newly prescribed at visit	51 (18.3)
Was on glaucoma medications before visit	228 (81.7)
REALM	
Eighth grade or lower	39 (14.0)
Ninth grade or higher	235 (84.2)
Age in years (mean ± SD)	65.8 ± 12.8

**Table 2:** Provider educates the patients about glaucoma medications by whether patients are prescribed glaucoma medications for the first time or if they were on them before the visit (*N* = 279).

Areas provider educated the patients about	Prescribed glaucoma medications for first time during the visit (*N* = 51) *N* (%)	Was on glaucoma medications before the visit (*N* = 228) *N* (%)	*P*
Adherence and adherence strategies	20 (39.2)	31 (13.6)	0.000
Amount/dose (number of drops)	8 (15.7)	8 (3.5)	0.001
Cost/insurance	6 (11.8)	17 (7.5)	0.309
Fear/concerns/barriers	4 (7.8)	7 (3.1)	0.123
Frequency of use	19 (37.3)	36 (15.8)	0.001
Generic/brand	4 (7.8)	21 (9.2)	0.760
How well medication is working	7 (13.7)	23 (10.1)	0.475
How to administer	13 (25.5)	27 (11.8)	0.014
Side effects	41 (80.4)	35 (15.4)	0.000
Importance of use	8 (15.7)	10 (4.4)	0.003
Last time used drops	Not applicable	2 (0.9)	0.498
Length of use	0 (0)	0 (0)	—
Name of medication	5 (9.8)	16 (7.0)	0.518
Nonglaucoma medications	5 (9.8)	18 (7.9)	0.681
Purpose	23 (45.1)	26 (11.4)	0.000
Supply	5 (9.8)	18 (7.9)	0.681
Which eye	17 (33.3)	13 (5.7)	0.000

**Table 3:** Provider educates the patients about glaucoma by whether patients are prescribed glaucoma medications for the first time or if they were on them before the visit (*N* = 279).

Areas provider educated about	Prescribed glaucoma medications for first time during the visit (*N* = 51) *N* (%)	Was on glaucoma medications before the visit (*N* = 228) *N* (%)	*P*
Physical changes that can occur with glaucoma and/or how to manage these changes	27 (52.9)	60 (26.3)	0.000
Emotional changes that can occur with glaucoma and/or how to manage these changes	0 (0)	1 (0.4)	0.633
Diagnosis	31 (60.8)	40 (17.5)	0.000
Family history	15 (29.4)	10 (4.4)	0.000
Goals of treatment	29 (56.9)	36 (15.8)	0.000
How to solve the problem	10 (19.6)	1 (0.4)	0.000
Intraocular pressure	29 (56.9)	106 (46.5)	0.219
Likelihood of long-term therapy	12 (23.5)	11 (4.8)	0.000
Management plan (ways to manage glaucoma without medications)	8 (15.7)	29 (12.7)	0.605
Prognosis	16 (31.4)	59 (25.9)	0.478

**Table 4:** Generalized estimating equation results predicting whether providers educate the patients about glaucoma and glaucoma medications (*N* = 279).

Independent variables	Education about glaucoma OR (95% CI)	*P*	Education about glaucoma medications OR (95% CI)	*P*
Patient age	0.98 (0.96, 1.01)	0.377	1.01 (0.98, 1.04)	0.056
Patient gender: female	1.06 (0.64, 1.76)	0.235	1.12 (0.68, 1.84)	0.474
Patient race: African American	0.47 (0.34, 0.66)	<0.001	1.67 (0.77, 3.62)	0.191
Newly prescribed glaucoma medications versus already on glaucoma medications	5.24 (2.05, 13.4)	0.001	7.26 (3.61, 14.6)	<0.001
REALM: reads at eighth grade level or less	0.93 (0.57, 1.51)	0.762	0.38 (0.18, 0.79)	0.009
Physician age	1.01 (0.97, 1.05)	0.742	1.04 (1.01, 1.07)	0.003
Physician gender-female	1.63 (0.70, 3.76)	0.257	2.24 (0.63, 7.9)	0.211

OR: odds ratio; 95% CI: 95% confidence interval.
